# HIV and STI Prevalence and Determinants among Male Migrant Workers in India

**DOI:** 10.1371/journal.pone.0043576

**Published:** 2012-08-27

**Authors:** Sangeeta S. Dave, Andrew Copas, John Richens, Richard G. White, Jayendrakumar K. Kosambiya, Vikas K. Desai, Judith M. Stephenson

**Affiliations:** 1 Institute for Women’s Health, University College London, London, United Kingdom; 2 Centre for Sexual Health & HIV Research, University College London, London, United Kingdom; 3 Department of Infectious Disease Epidemiology, London School of Hygiene & Tropical Medicine, London, United Kingdom; 4 Department of Community Medicine, Government Medical College, Surat, Gujarat, India; Tulane University, United States of America

## Abstract

**Background:**

Our objective was to estimate for the first time the prevalence and determinants of human immunodeficiency virus type 1 (HIV-1) and sexually transmitted infections (STIs) among male migrants in India.

**Methodology/Principal Findings:**

We conducted a multi-stage stratified probability sample survey of migrant (defined as not born in Surat city) men aged 18 to 49 years working in the diamond and textile industries in Surat city. Behavioural and biological data were collected. Biological data included laboratory diagnosed *herpes simplex virus type 2* (HSV-2), syphilis, chlamydia, gonorrhoea, *Trichomonas vaginalis* (together defined as ‘any STI’) and HIV-1. Likely recently acquired STIs included chlamydia, gonorrhoea, *T.vaginalis* and syphilis with rapid plasma reagin ≥1∶8. The response rate was 77% (845/1099). Among 841 participants, HIV-1 prevalence was 1.0%, ‘any STI’ prevalence was 9.5% and 38.9% of these STIs were likely to have been recently acquired. Being a diamond worker, Surat resident for 10+ years and recent antibiotic use were each associated with higher odds of ‘any STI’ (aORs 1.83 (95% CI 1.09–3.09), 1.98 (95% CI 1.22–3.22) and 2.57 (95% CI 1 .17–5.64), respectively) after adjusting for the other two factors and age. The main study limitation was social desirability bias for self-reported sexual behaviour; STIs were diagnosed in some self-reported virgins.

**Conclusions/Significance:**

HIV and STI prevalence were lower than expected, but prevention interventions remain necessary in Surat since almost 40% of STIs among participants were probably recently acquired and sentinel surveillance HIV prevalence remains high. The participants had a similar HIV prevalence to Surat antenatal clinic attendees, a proxy for the general population. This suggests migrants are not always at higher risk of HIV compared to the general population in their migration destination. Our findings highlight the need to contextualise research findings from a specific setting with other local information to guide HIV/STI prevention interventions.

## Introduction

In India 2.4 million people are estimated to have the human immunodeficiency virus (HIV) [1] and there is a lack of reliable sexually transmitted infection (STI) incidence and prevalence data for India. However the Indian National AIDS Control Organisation (NACO) has estimated the annual STI incidence in India at 5%, equivalent to 40 million new cases each year [2]. Groups perceived to have higher risk sexual behaviour are targeted with HIV and STI prevention interventions under NACO’s National AIDS Control Programme (NACP).

Movement in search of work is common in India and other developing countries. The current NACP is focused on migrants, especially single male migrants, who are perceived to be at high risk of HIV and STI infection and are therefore an important bridging group for the transmission of these infections from core groups to the general population [3]. However the majority of data on male migrants are from sub-Saharan Africa [4], [5], [6], [7], [8] and there is a lack of data from within India to guide national prevention programmes.

Here we present the first estimates of the prevalence and determinants of HIV infection among male migrants in India. The data are from a survey of migrant men aged 18 to 49 years conducted between 2005 and 2006 in the diamond and textile industries in the highly industrialised city of Surat in western India. We chose these industries since they are the two main industries in Surat and the majority of employees are migrant men. In 2005 the city had a high HIV sentinel surveillance prevalence (>5% among STI clinic attendees or high risk groups and >1% among antenatal clinic (ANC) attendees). It is situated in Gujarat, a medium HIV prevalence state (>5% among STI clinic attendees or high risk groups and <1% among ANC attendees). The high risk groups are comprised of female sex workers and men who have sex with men [9]. At the time of the study, local non-governmental organisations (NGOs) carried out workplace based HIV/STI prevention interventions during which they provided information and education about HIV and STIs and their prevention and distributed condoms.

## Materials and Methods

### Study Design

Multi-stage stratified probability sample survey.

### Sampling Frame

In 2005, textiles were sold through 120 markets employing an estimated 130,000 men. The diamond industry employed approximately 500,000 men working in 20,000 diamond units housed in larger buildings known as complexes. We selected and mapped four diamond sub-areas and 19 textile markets which had received partial HIV intervention coverage including HIV and STI information, education and communication work and condom distribution by NGOs a year prior to the study start date. The sampling frame consisted of 226 diamond complexes and 19 textile markets.

### Sampling

We randomly selected 41 diamond complexes and 11 textile markets from the sampling frame. Diamond complexes were stratified into four strata according to size. The largest stratum consisted of the single largest complex sampled with certainty. Complexes were randomly selected within the remaining three strata, to give a total of forty. Units were randomly selected with replacement from each complex with probability proportional to size. The number of workers sampled from each unit varied across strata; five for stratum one [smallest complexes], ten for stratum two, 20 for stratum three and 30 for stratum four [largest complex]. These numbers were selected taking into account the size and number of complexes from each stratum to ensure that the probability of selection for any diamond worker was roughly equal.

The eight largest markets were sampled with certainty and three were randomly selected from the remaining markets with probability proportional to size. The total number of men sampled from the markets was proportional to market size amongst the eight largest markets and fixed in the smaller three sampled markets. Within each market workers were stratified by one of ten occupation groups and selected proportional to the size of the stratum. Within both industries workers were selected from workplaces by systematic sampling. Selection was without substitution if a migrant man refused to participate. If a non-migrant man was selected, other men were systematically approached until a migrant worker was found.

### Eligibility

Men aged 18 to 49 years were eligible to participate if they had not been born in Surat city.

### Sample Size

A target effective sample size of 456 men was required to estimate an expected HIV-1 point prevalence of 5%, i.e. a 95% CI 3% to 7%, within 2%. We assumed a design effect of 1.45, based on an average cluster size of 10 workers and an intracluster correlation of 0.05, and a participation rate of 60%. We therefore aimed to approach 1100 men to recruit 661 participants.

### Questionnaires

We developed three standardized interviewer-administered questionnaires in English and two local languages. These included a sexual behaviour and HIV/STI knowledge, attitudes and perceptions questionnaire, and clinical history and examination questionnaires. Sexual behaviour data included lifetime sexual behaviour, and three most recent female and male sexual partnerships and partner types in the past year. Clinical data included STI treatment history and symptoms and signs related to HIV and STIs. Data collected on non-responders included industry, age, place of birth, marital status including whether or not married men were living with their wives.

### Consent

We obtained written informed consent from literate participants and witnessed informed verbal consent from illiterate participants in their language.

### Data Collection

A unique study number was used to link questionnaires with laboratory samples. Six interviewers collected demographic and sexual behaviour data and six doctors collected clinical data. Participants were interviewed by study staff at government and private health centres out of hours. Laboratory staff collected the biological samples. Each participant was given a torch as a gift after participation.

### Biological Samples

We collected blood samples to test for HIV-1, *Herpes Simplex Virus types 2* (HSV-2), and *Treponema pallidum* (syphilis) and first catch urine samples to test for *Chlamydia trachomatis*, *Neisseria gonorrhoea* and *Trichomonas vaginalis*. Participants were given all the results except HIV and offered free HIV counselling and testing at local government facilities.

**Figure 1:**
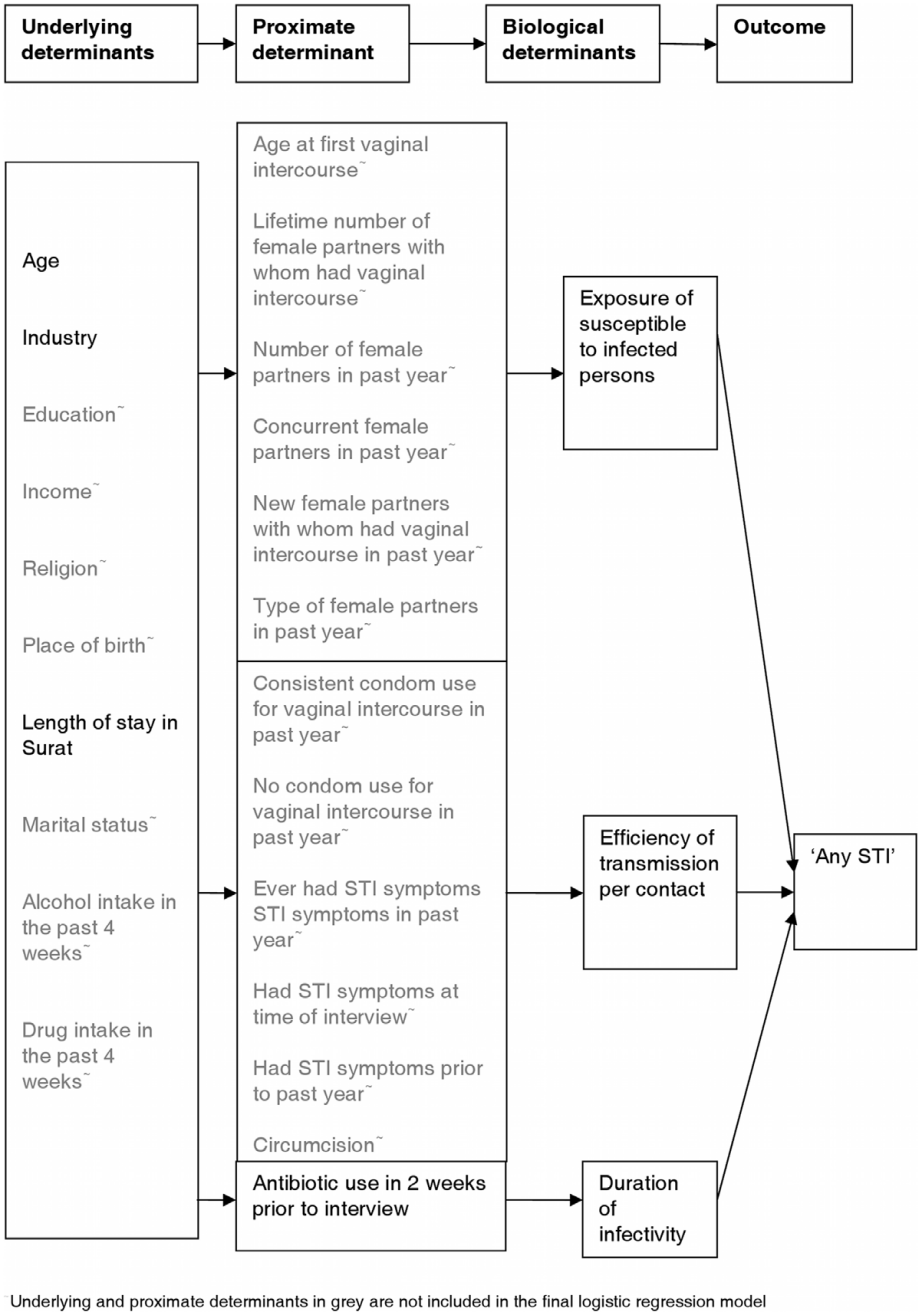
Final conceptual determinants model for ‘any STI’. A four level conceptual determinant model was developed for any prevalent STI diagnosis on the day of interview. The model is based on the relationship between the biological determinants of STI infection and their effect on STI outcomes. *Ro = BcD* summarises the relationship of the biological determinants of STI infection where *Ro* is the reproductive number of an infection defined as the average number of secondary cases which arise from a new case of infection, *B* is the efficiency of transmission per contact, *c* the exposure of susceptible persons to infected persons and *D* the duration of infectivity. The model has four levels: underlying determinants, proximate determinants, biological determinants and the outcome, ‘any STI’. The underlying determinants act through the proximate determinants to influence the biological determinants which in turn determine the outcome ‘any STI’. The underlying determinants included socio-demographic, economic and lifestyle factors. The proximate determinants included sexual behaviour and reported STI symptom related factors including treatment, and circumcision. Figure 1 represents the determinants included in the final logistic regression model for ‘any STI’. This model only included determinants which were associated with ‘any STI’ at a significance level of p<0.05. Determinants not included in the final logistic regression model are shown in grey.

### Laboratory Testing

We tested for HIV-1 with two methodologically different enzyme-linked immunosorbent assays (ELISAs) (J Mitra Microlisa HIV EIA (HIV subgroup O & subgroup C) and Genedia 1/2 Elisa 3.0) and used an enzyme immunoassay (EIA) for indeterminate or minimally reactive results (PBS Orgenics EIA Immunocomb II HIV 1 & 2 Bispot). We tested all serum samples for syphilis with rapid plasma reagin (RPR) and treponema pallidum haemagglutination (TPHA) (Omega Diagnostics Immutrep). All positive RPR samples were confirmed with a different RPR (Plasmatec). Western blots were used to confirm syphilis if a result was TPHA positive but RPR negative (Euroimmune TP IgM and IgG). Active syphilis (primary and secondary syphilis) was defined as RPR titre ≥1∶8 [10]. We used IgG and IgM ELISAs to test for HSV-2 (Philco) and cultured *T.vaginalis* (TV In-pouch). Polymerase chain reaction (Roche amplicor) was used to detect chlamydia and gonorrhoea in urine sample aliquots pooled into fives. If a pool tested negative, all samples were reported as negative. If it tested positive, a second aliquot of each sample in the pool was tested individually. The validity of this approach is well established [11], [12].

Processed samples were stored between 2 to 8°C at study sites and during transportation and at −20°C at the storage centre in Surat. They were transported either on the day of collection or the following day to Biocare Laboratories Ltd., Ahmedabad for HIV-1, HSV-2, syphilis and *T.vaginalis* testing. Urine sample aliquots were sent weekly to Auroprobe Laboratories, New Delhi for chlamydia and gonorrhoea testing. All samples were stored at −80°C except for *T.vaginalis* which was kept at room temperature up to 37°C until the final report at five days.

Ten percent of each of the following samples, HIV-1, HSV-2 IgM and IgG, RPR and TPHA including all positive samples and randomly selected negative samples were sent for external quality control (EQC). All positive chlamydia and gonorrhoea samples and 2% of negative samples also had EQC. There was good agreement between EQC and study results (HIV-1, CT and NG 100%, RPR and TPHA 97.6%, HSV-2 IgM 94% and HSV-2 IgG 92.9%). *T.vaginalis* samples were not sent for EQC due to poor survival beyond five days.

### Statistical Methods

#### Double-entry for data

Data were double-entered and verified using Epi Info 6 software.

#### Outcomes

The outcomes were HIV-1 and ‘any STI’. ‘Any STI’ was defined as chlamydia, gonorrhoea, *T. vaginalis,* HSV-2 or syphilis diagnosed positive on laboratory testing.

#### Determinants

The determinants included socio-demographic factors and sexual behaviour for both HIV-1 and ‘any STIs’, and also reported and laboratory diagnosed STIs for HIV-1. Laboratory diagnosed STIs were grouped into likely recently acquired STIs (active syphilis (RPR≥1∶8), chlamydia, gonorrhoea, *T.vaginalis*), ulcerative STIs (serologically diagnosed herpes simplex virus 2 and syphilis) and non-ulcerative STIs (chlamydia, gonorrhoea and *Trichomonas vaginalis*).

#### Effect measures

We calculated prevalence, unadjusted and adjusted odds ratios (OR). Only % and p values were calculated for rare determinants, i.e. less than 5% of participants, and the p values obtained using a survey command were confirmed with Fishers’ exact test.

**Table 1:** Socio-demographic characteristics, sexual behaviour, STI symptoms, antibiotic use and self-reported circumcision status of participants by industry.

				Diamond	Textile	
		N^a^	nb	n^c^	n^c^	p value
		845		455	390	
***Socio-demographic characteristics***						
				% (95% CI)^d^	% (95% CI)^d^	
**Age (years)**	18–24		363	43.3 (37.9–49)	44.5 (39.8–49.3)	0.06
	25–29		200	25.1 (21.5–29.2)	22.2 (18.3–26.6)	
	30–34		128	16.9 (13.7–20.5)	12.3 (9.6–15.8)	
	35–49		154	14.7 (10.7–19.7)	21.0 (17.5–25.0)	
**Place of birth**	Within Gujarat		384	78.0 (71.5–83.3)	7.3 (5.2–10.1)	<0.001
	Other state in India		461	22.0 (16.7–28.5)	92.7 (89.9–94.8)	
**Length of stay in**	≤4		246	36.5 (32.2–41.1)	23.8 (19.8–28.3)	<0.001
**Surat (years)**	5 to 9		211	25.5 (21.3–30.1)	27.6 (23.4–32.1)	
	10 to 14		193	16.7 (13.6–20.4)	27.7 (23.5–32.3)	
	15 to 19		105	10.7 (8.3–13.7)	12.8 (9.8–16.7)	
	20 to 36		87	10.7 (7.3–15.3)	8.2 (5.9–11.1)	
	*missing data*		*3*			
**Education**	Illiterate		124	13.3 (10–17.6)	15.7 (12.5–19.5)	0.07
	Primary school		281	35.0 (30.5–39.7)	31.1 (26.8–35.9)	
	Secondary school		344	43.1 (38.4–48.1)	39.1 (34.4–44.0)	
	Higher education		94	8.6 (6.0–12.2)	14.1 (11.0–17.9)	
	*missing data*		*2*			
**Monthly income**	≤2500		150	4.7 (2.8–7.6)	33.6 (28.9–38.7)	<0.001
**(rupees)**	2501 to 3500		239	12.8 (9.8–16.6)	45.9 (41.2–50.8)	
	3500 to 5000		259	46.1 (41.5–50.8)	12.6 (9.1–17.3)	
	>5000		196	36.4 (30.5–42.7)	7.9 (5.7–10.7)	
	*missing data*		*1*			
**Religion**	Hinduism		801	98.9 (96.9–99.6)	90.5 (87.5–92.9)	<0.001
	Other^*^		42	1.1 (0.4–3.1)	9.5 (7.1–12.5)	
	*missing data*		*2*		*0*	
**Alcohol consumption**	Yes		129	9.3 (6.9–12.5)	22.2 (18.5–26.5)	<0.001
**in previous month**	No		712	90.7 (87.5–93.1)	77.8 (73.5–81.6)	
	*missing data*		*4*			
**Illicit drug use in**	Yes		17	0.5 (0.1–1.8)	3.8 (2.3–6.1)	<0.001
**previous month**	No		828	99.6 (98.2–99.9)	96.2 (93.9–97.7)	
**Marital status**	Married living with wife		320	39.8 (34.3–45.7)	34.2 (29.9–38.7)	0.001
	Married not living with wife		223	20.6 (16–26)	33.6 (29–38.6)	
	Unmarried		300	39.6 (33.7–45.8)	32.2 (27.4–37.4)	
	*missing data*		*2*			
**STI symptoms on day**	Yes		35	4.0 (2.7–5.9)	4.3 (2.8–6.7)	0.81
**of interview**	No		806	96.0 (94.1–97.3)	95.7 (93.3–97.3)	
	*missing data*		*4*			
**Antibiotic use in**	Yes		27	5.3 (3.3–8.5)	0.8 (0.3–2.5)	<0.001
**previous 2 weeks**	No		814	94.7 (91.6–96.7)	99.2 (97.5–99.7)	
	*missing data*		*4*			
**Self-reported**	Yes		39	1.5 (0.8–3.0)	8.1 (6.0–11.0)	<0.001
**circumcision**	No		802	98.5 (97.0–99.2)	91.9 (89.0–94.0)	
	*missing data*		*4*			
***Sexual behaviour***						
**Ever had sex with**	Yes		665	74.7 (70.4–78.6)	83.1 (79.0–86.5)	<0.01
**male and/or**	No		179	25.3 (21.4–29.6)	16.9 (13.5–21.0)	
**female partners^e^**	*missing data*		*1*			
**Ever had vaginal**	Yes		654	74.1 (69.4–78.4)	80.8 (76.6–84.4)	0.03
**intercourse**	No		190	25.9 (21.7–30.6)	19.2 (15.6–23.4)	
	*missing data*		*1*			
**Age at first vaginal**	8–15		71	10.1 (7.1–14.2)	12.0 (9.0–15.9)	0.38
**intercourse in years**	16–19		225	32.9 (27.3–39.0)	36.7 (31.2–42.5)	
	20–35		357	57.1 (51.0–62.9)	51.3 (45.5–57.2)	
	*missing data*		*1*			
**Number of lifetime**						<0.001
**female partners with**	1		417	68.1 (62.5–73.2)	59.0 (53.6–64.2)	
**whom had vaginal**	2		130	21.0 (17.0–25.6)	19.0 (14.9–23.8)	
**intercourse**	≥3		107	10.9 (7.9–14.9)	22.1 (18.0–26.8)	
**Had vaginal**						
**intercourse in past**	Yes		590	91.0 (87.3–93.7)	89.0 (84.9–92.0)	0.40
**year**	No		64	9.02 (6.4–12.7)	11.0 (8.0–15.1)	
**Consistent condom**						0.38
**use for vaginal**	Yes		150	23.9 (17.9–31.2)	28.0 (22.3–34.5)	
**intercourse in past**	No		439	76.1 (68.8–82.1)	72.0 (65.5–77.7)	
**year**	*missing data*		*1*			
**Types of female**		523				0.04
**partner in**	Wife only		483	94.8 (91.2–97.0)	89.9 (85.7–93.0)	
**past year if married**	Wife & other female partners		39	5.2 (3.0–8.8)	10.1 (7.0–14.3)	
	*missing data*		*1*			

N^ a^ is the total unweighted denominator per determinant. It was 845, the total number of participants in the study, for all the following variables: socio-demographic characteristics, STI symptoms, antibiotic use, self-reported circumcision status and ever had sex with male of female partners. N was 654, the number of men who reported ever having had vaginal intercourse for the determinants age at first vaginal intercourse in years, the number of lifetime female partners with whom had vaginal intercourse and whether or not participants had vaginal intercourse in the past year. For the determinant consistent condom use for vaginal intercourse in past year, N was 590 which was the number of men who had vaginal intercourse in the past year. For the determinant type of female partner in the past year, N was 523, the number of married participants in the study who had vaginal intercourse in the past year. n^b^ is the unweighted number of participants by variable category. n^c^ is the total unweighted number of participants by industry. ^d^ is the weighted column % and 95% confidence interval for participants by industry for each category of each determinant.^ e^Ever had sex with male and/or female partners includes vaginal, oral, or anal intercourse. ^*^Other religion includes Islam, Buddhism and Jainism.

#### Statistical analysis

We used Stata v.8 for data analysis. The survey commands in STATA were used to take stratification, clustering and weighting into account. For the diamond industry the first randomly selected sampling unit (primary sampling unit or PSU) was each person in the largest stratum and each complex in the remaining strata. For the eight largest textile markets, strata were defined by job type and PSU was defined as the participants recruited to the study. The three smallest markets together made up one stratum with PSU defined by each individual market. To take into account significant differences between diamond participants and non-responders in the length of time men had lived in Surat and whether or not married men were living with their wives in Surat and between textile participants and non-responders in marital status we weighted the participant data prior to analysis to increase the generalisability of the results to the study population.

#### Regression analysis for a diagnosis of ‘any STI’

The outcome for regression analysis was ‘any STI’ since the prevalence of individual STIs was relatively low. So that the study was adequately powered to detect associations between the determinants and ‘any STI’ and since there were no significant interactions between industry and the determinants, data for both industries were combined prior to further analysis.

Underlying determinants of STIs can be used to identify populations for HIV/STI prevention interventions. We developed a conceptual framework (Figure 1) similar to that for HIV [13]. The model is based on the relationship between the biological determinants of STI infection and their effect on STI outcomes. Underlying determinants act through proximate determinants to influence biological determinants which in turn determine the STI outcome. Two model selection procedures were performed based on logistic regression. A base model was built from a forward stepwise model selection procedure of proximate determinants, using a significance level of p<0.05. A full model resulted from selecting further underlying determinants significant at p<0.05 and added to the base model. Underlying determinants had a higher threshold for inclusion in the full model compared to the proximate determinants, as only those which could not be fully explained by proximate determinants were retained in the full model. Evidence for interaction between underlying and proximate determinants was explored separately for each model.

**Table 2:** Prevalence of HIV-1 infection and STIs among participants by industry.

	Diamond	Textile		All participants	
	% (95% CI)	% (95% CI)	p value	% (95% CI)	N^a^
**HIV 1**	0.9 (0.3–2.8)	1.0 (0.4–2.6)	0.91	1.0 (0.5–2.0)	841
**Any STI excluding HIV**	12 (9.1–15.6)	7.0 (4.7–10.3)	0.02	9.5 (7.5–11.9)	841
**Non-ulcerative STI** c	4.3 (2.8–6.4)	1.9 (0.9–3.9)	0.05	3.1 (2.1–4.4)	840^b^
**Ulcerative STI^d^**	8.1 (5.7–11.4)	5.4 (3.5–8.2)	0.14	6.7 (5.1–8.8)	841
**Recently acquired STI** e	4.5 (3.0–6.7)	2.9 (1.6–5.2)	0.22	3.7 (2.6–5.2)	841
**Chlamydia**	0.6 (0.2–1.7)	0.8 (0.3–2.5)	0.73	0.7 (0.3–1.6)	840^b^
**Gonorrhoea**	1.5 (0.7–3.2)	0.2 (0.04–1.8)	0.05	0.9 (0.4–1.8)	840^b^
**Trichomonas vaginalis**	2.1 (1.0–4.4)	0.8 (0.3–2.5)	0.14	1.5 (0.8–2.7)	840^b^
**Herpes simplex virus 2**	3.3 (2.0–5.4)	3.5 (2.1–5.9)	0.83	3.4 (2.4–4.9)	841
**Syphilis**	5.3 (3.4–8.1)	2.3 (1.2–4.5)	0.04	3.8 (2.6–5.5)	841
**Active syphilis** f	0.2 (0.03–1.3)	1.0 (0.4–2.7)	0.09	0.6 (0.3–1.5)	841

N^a^ is unweighted denominator of men who provided a blood sample.

b unweighted denominator of men who provided a urine sample.

c Non-ulcerative STI includes chlamydia, gonorrhoea and *Trichomonas vaginalis*. ^d^Ulcerative STI includes Herpes simplex virus 2 and syphilis.

e Likely recently acquired STI includes active syphilis, chlamydia, gonorrhoea and *Trichomonas vaginalis*.

f Active syphilis if RPR titre ≥1∶8.

**Table 3:** Association of determinants with HIV-1 infection.

		HIV-1 infection			
		%	(n/N)^a^	p value	base^b^
Age in years	18–24	0	(0/361)		841
	25–29	2.4	(5/199)		
	30–49	1.2	(3/281)	0.03 (<0.001)^c^	
Education	Higher	0.7	(3/434)		839
	Primary	1.7	(5/281)		
	Illiterate	0	(0/124)	0.26 (0.27)^c^	
Monthly income in rupees	>3500	0.99	(4/454)		840
	≤3500	0.92	(4/386)	0.92	
Length of stay in Surat in years	<5	0.4	(1/244)		838
	5–9	0.5	(1/210)		
	10–36	1.6	(6/384)	0.33	
Industry	Diamond	0.9	(4/452)		841
	Textile	1.0	(4/389)	0.92	
Marital status	Living with wife	1.9	(6/320)		840
	Not living with wife	0.9	(2/222)		
	Unmarried	0	(0/298)	0.08 (0.04)^c^	
History of circumcision	No	1.0	(8/800)		839
	Yes	0	(0/39)	0.56 (1.00)^d^	
Any STI	No	0.5	(4/758)		841
	Yes	5.0	(4/83)	<0.001	
Any ulcerative STI	No	0.5	(4/782)		841
	Yes	7.1	(4/59)	<0.001	
Any non-ulcerative STI	No	0.9	(7/813)		840
	Yes	4.1	(1/27)	0.16	
Ever had sex with male and/or female	No	0	(0/177)		841
partners^e^	Yes	1.2	(8/664)	0.17	
Age at first vaginal intercourse in years^f^	8–15	0.8	(3/356)		652
	16–19	2.9	(2/71)		
	20–35	1.4	(3/225)	0.41	
Number of lifetime female partners	1	0.7	(3/416)		653
with whom had vaginal intercourse^f^	2	1.6	(2/130)		
	≥3	2.9	(3/107)	0.28	
Consistent condom use in the past year^f^	No	1.5	(8/552)		588
for vaginal intercourse	Yes	0	(0/36)	0.48 (1.00)^d^	
					

a N Unweighted denominator is the number of participants per determinant category. n is the number of participants per determinant category who were diagnosed with HIV-1 infection.

b Unweighted base is the total number of all participants per determinant. It varies due to item non-response and the number of participants per determinant who reported ever having had sex (vaginal, or oral, or anal intercourse) with female partners or in the past year with up to three female partners.

c p value in () by variant of Fisher’s exact test.

d p value in () by Fisher’s exact test.

e Ever had sex with male and/or female partners includes vaginal, or oral, or anal intercourse.

f These data are only for participants who reported vaginal intercourse.

## Results

### Comparison of Survey Participants and Non-responders

The overall participation rate was 77% (845/1099). Diamond workers had a higher response compared to textile workers (83.2%, 456/548 vs. 70.8%, 390/551). Diamond participants and non-responders were similar with respect to birthplace (Gujarat excluding Surat 78.2% vs. 69.7%, respectively), median age (both 26 years) and marital status (married: both 59%). However diamond participants reported a significantly longer stay in Surat (median 8 years vs. 5 years, p = 0.01) than non-responders and if married, were significantly more likely to report living with wives than non-responders (67.7% vs. 41.9%, p<0.01). Textile industry participants and non-responders were similar in age (median 26 years vs. 24 years, respectively), birthplace (Indian state other than Gujarat: 92.8% vs. 95.0%, respectively) length of stay in Surat (median 9 years vs. 8 years, respectively), and reporting they lived with wives (50.2% vs. 53.5%). Textile participants were significantly more likely to report they were married compared to non-responders (70.5% vs. 61.5%, p = 0.04).

**Table 4:** Unadjusted association of determinants with ‘any STI’*.

		Any STI					
		%	(n/N)^a^	OR	(95% CI)	p value	base^b^
Age in years	18–24	8.1	(30/361)	1.00			841
	25–29	6.1	(13/199)	0.73	(0.39–1.38)	0.03	
	30–49	13.8	(40/281)	1.81	(1.09–3.00)		
Education	Higher	9.7	(45/434)	1.00			839
	Primary	9.4	(26/281)	0.97	(0.60–1.56)	0.96	
	Illiterate	9.0	(12/124)	0.92	(0.49–1.71)		
Monthly income	>3500	10.6	(50/454)	1.00			840
in rupees	≤3500	8.1	(32/386)	0.74	(0.47–1.17)	0.20	
Length of stay in	<5	9.3	(23/244)	1.00			838
Surat in years	5–9	5.1	(11/210)	0.53	(0.26–1.06)	0.04	
	10–36	12.3	(49/384)	1.36	(0.84–2.20)		
Industry	Diamond	12.0	(56/452)	1.00		0.03	841
	Textile	7.0	(27/389)	0.55	(0.33–0.93)		
Marital status	Living with wife	10.0	(33/320)	1.00			840
	Not living with wife	7.5	(18/222)	0.73	(0.42–1.25)	0.44	
	Unmarried	10.1	(31/298)	1.00	(0.62–1.64)		
STI symptoms on day	No	9.4	(79/804)	1.00			839
of interview	Yes	8.0	(3/35)	0.84	(0.24–2.94)	0.78	
Antibiotic use in the 2	No	9.0	(76/812)	1.00			839
weeks prior to interview	Yes	22.3	(6/27)	2.91	(1.33–6.39)	<0.001	
History of circumcision	No	9.8	(81/800)	1.00			839
	Yes	2.1	(1/39)	0.20	(0.03–1.39)	0.10	
Ever had sex with male	No	9.8	(18/177)	1.05	(0.61–1.84)		841
and/or female partners∼	Yes	9.4	(65/664)	1.00		0.86	
Age at first vaginal	8–15	13.0	(9/71)	1.45	(0.67–3.13)	0.45	652
intercourse in years^d^	16–19	8.4	(20/225)	0.89	(0.50–1.56)		
	20–35	9.4	(35/356)	1.00			
Number of lifetime female	1	7.7	(34/416)	1.00			653
partners with whom had	2	11.8	(16/130)	1.60	(0.70–3.64)	0.21	
vaginal intercourse^d^	≥3	13	(14/107)	1.79	(0.92–3.47)		
Type of partner in last year ifmarried^d^	Wife only	8.5	(43/482)	1.00		0.22	521^c^
	Wife & other female partners	14.7	(6/39)	1.87	(0.70–5.01)		
Consistent condom use	No	9.5	(55/552)	1.00			588
in the past year^d^	Yes	8.6	(3/36)	0.89	(0.27–3.00)	0.86	

* STIs: syphilis, herpes simplex virus, gonorrhoea, chlamydia & *Trichomonas vaginalis*.

a N Unweighted denominator is number of participants per determinant category. n is number of participants per determinant category diagnosed with a STI.

b Unweighted base is the total number of all participants per determinant.

c Unweighted base is the total number of married participants per determinant. It varies due to item non-response and the number of participants per determinant who reported ever having had sex (vaginal, or oral, or anal intercourse) with female partners or in the past year with up to three female partners.

d For participants who reported having sex.

∼ Ever had sex with male and/or female partners includes vaginal, or oral, or anal intercourse.

**Table 5:** Final model of adjusted ORs for the association of determinants with ‘any STI’*.

		aOR (95% CI)∼	p value
**Final model**			
Antibiotic use in 2 weeks	No	1	0.02
prior to interview	Yes	2.57 (1.17–5.64)	
Industry	Textile	1	
	Diamond	1.83 (1.09–3.09)	0.03
Length of stay in	<10	1	
Surat in years	≥10	1.98 (1.22–3.22)	<0.001

∼ Adjusted for all variables in the table and age.

* STIs: syphilis, herpes simplex virus, gonorrhoea, chlamydia & *Trichomonas vaginalis*.

### Survey Completion, Missing Data and Inter-observer Variability

Of 845 participants, 839 (99.3%) men completed the survey. There were few missing data (range 0.2% to 6.3% across items). Four men declined to provide biological samples. There was a high level of agreement between study doctors in their diagnoses of genital discharge, ulcers and circumcision and among study interviewers with respect to their documentation of participants’ responses to seven sexual behaviour questions. We excluded data from one diamond worker who only gave samples, and report the findings for the remaining 455 diamond and 390 textile workers.

### Socio-demographic Characteristics, STI Symptoms, Sexual Behaviour, Self-reported Circumcision and Antibiotic Use

Compared to diamond workers, textile workers were significantly more likely to report they were born outside Gujarat, had lived in Surat for longer, were not living with their wives, had lower incomes and were not Hindus (Table 1). Self-reported circumcision and recent alcohol consumption, illicit drug use, antibiotic use and STI symptoms on the day of interview were uncommon. Textile workers were significantly more likely to report they had ever been sexually active, had ever had vaginal intercourse and had more than one lifetime female partner compared to diamond workers. Among married men, textile workers were significantly more likely to report female partners apart from their wife in the past year.

### HIV-1 Infection

Eight participants were diagnosed with HIV-1, (see Table 2). Age and any STI were significantly associated with HIV-1 (see Table 3). Men with any STI and those with serological syphilis or HSV-2 were far more likely to be diagnosed with HIV-1 compared to men without STIs. There were no infections among men aged less than 25 years, unmarried men, those who reported consistent condom use in the past year or a history of circumcision. Most HIV diagnoses were in men who were living with their wives and had lived in Surat for more than ten years. We did not analyse HIV-1 further since few men had the infection.

### STI Infection

We diagnosed STIs in 9.5% of participants (see Table 2). Of these, 38.9% were likely recently acquired STIs. The two most common diagnoses, syphilis (3.8%) and HSV-2 (3.4%) accounted for 71% of all STIs although none of the participants were noted to have genital ulceration on examination. Chlamydia and gonorrhoea were both uncommon (0.7% and 0.9%, respectively). Diamond workers were almost twice as likely to be diagnosed with any STI and the most common STI, syphilis, compared to textile workers. Few syphilis diagnoses were due to active syphilis (16%). Of eighty-three men diagnosed with STIs, 21.7% (nine syphilis, six trichomonas, one chlamydia, one gonorrhoea and one HSV-2) reported they were virgins. STIs were diagnosed in similar proportions of men who reported ever having had sex and never having had sex (9.4% of 664 men and 9.8% of 177 men).

Age, length of stay in Surat, industry and antibiotic use were significantly associated with having ‘any STI’ in unadjusted analysis (see Table 4). We also analysed our data using a lower cut-off of two years stay in Surat based on a recent survey among male migrants in India [14]. Living in Surat for more or less than two years was not associated with either having HIV-1 or any STI (1.0% vs. 0.8%, p = 0.87 and 9.0% vs. 12.1%, p = 0.21, respectively).

We included all participants in the multivariate analysis since sexual behaviours were not significantly associated with having any STI. The base model consisted of antibiotic use in the two weeks prior to interview. The final model included antibiotic use, length of stay in Surat and industry. After adjusting for other factors, men who had used antibiotics in the two weeks prior to interview, had lived in Surat for at least ten years or were diamond workers had significantly increased odds of having any STI (aOR 2.57, aOR 1.98 and aOR 1.83, respectively) (see Table 5).

## Discussion

The study participants had a much lower than expected HIV-1 prevalence of 1.0% and a STI prevalence of 9.5%, arising from a mixture of acute and non-acute infections, and reflecting both recent and lifetime risk. Diamond work, living in Surat for at least 10 years and antibiotic use in the two weeks prior to interview were each associated with higher adjusted odds of having an STI; antibiotic use had the strongest association (aOR 2.57).

There were some socio-demographic differences between men from the two industries in that textile workers were more likely to report lifetime sexual activity, multiple female partners and among married men non-spousal partners in the past year whereas diamond workers were more likely to have an STI and report recent antibiotic use. However, as described in the methods, analysis of the determinants of ‘any STI’ was based on combined data from the two industries because interaction testing revealed no significant evidence that associations with determinants differ between industries.

The low HIV-1 and STI prevalence may in part be explained by most participants reporting only one sexual partner in their lifetime (78.9%). In the majority of cases this was a spouse and men who were more than 25 years old were much more likely to be married than younger men. The association between longer stay in Surat and STIs, after adjusting for age, is consistent with an increase in the rate of STI acquisition on migration to Surat. However the lack of overlap of STIs in individuals suggests that many of the sexual encounters may be low risk events. It is unclear why having an STI was associated with antibiotic use in the two weeks prior to interview and may be due to chance. This is especially likely given that past syphilis and HSV-2 over a lifetime account for the highest prevalence of “any STI”. In addition there was no association between STIs and self-reported STI symptoms on the day of interview.

The strengths of our study include HIV-1 and STI results verified by EQC, a high participation rate, low item non-response and low HIV or STI testing refusals. The limitations include the cross-sectional study design which limited our ability to interpret causal associations, social desirability bias and STI diagnosis.

STI diagnoses in self-reported virgins in the study are likely to reflect social desirability bias for self-reported sexual behaviour. This is supported by the data from focus groups we conducted with 37 migrant men in Surat’s diamond and textile industries in 2007 in which they discussed the stigma faced by men who had pre- or extra-marital sex. The method by which we obtained the sexual behaviour data, face to face interviews (FTFI), may also have contributed to this bias. In our study, two Indian national general population surveys and a recent Indian sexual behaviour survey which used FTFIs, sex with female sex workers (FSWs) in the past year was reported by 2.4%, <1%, 3.4% and 2%, respectively [15], [16], [17]. However in the latter study when a more anonymous method, polling booth surveys, was used 11% of participants reported sex with FSWs.

Some of the STIs, syphilis, HSV-2 and gonorrhoea, found in self-reported virgins may be due to false positive laboratory test results. This can be a result of cross-reactivity of *N.gonorrhoeae* with non-pathogenic Neisseria species and of HSV-2 IgG with HSV-1 IgG [18]. RPR biological false positives can occur with other conditions and RPR sensitivity can vary between 44% and 76% in primary syphilis and between 70% and 73% for late latent or late syphilis [19]. Therefore all positive RPRs were confirmed by a different manufacturer’s RPR test and TPHA and Western Blot were used as described in the methods. Some of the syphilis may have been either acquired congenitally or through blood transfusions; HIV-1 may also have been acquired through blood transfusions.

The study sample may have been at lower risk of HIV and STI acquisition than the study population since within the textile industry participants were more likely to be married than non-participants and diamond industry participants were more likely to live with their wives than non-participants. To try and take these differences into account and make our results more generalisable to the study population, we weighted on marital status for the whole study sample and on duration of stay in Surat for diamond industry participants since they differed in this from non-responders. However this may not have corrected for systematic differences in sexual risk behaviour and the results should therefore be treated with a degree of circumspection.

On comparison of our results with those of other studies of male migrants from less developed countries, HIV prevalence was higher among Nepalese migrants who had worked in India (10.3%) [20] and considerably higher among migrants within Africa (range 24.4% and 33.9%) [4], [5], [6], [7], [8]. Migrants within Pakistan and China also had low gonorrhoea and chlamydia prevalence similar to the study participants (Pakistan, 0.5% vs. 1% and China, 0% to 3.5% vs. 1.8% to 0.5%, respectively) [21], [22], [23]. Nepalese migrants had a higher syphilis prevalence (25%) compared to the study participants and Chinese migrants (1%) [20], [22]. HSV-2 prevalence was higher among Chinese migrants (5.5%) [24] compared to the study participants. As in our study, longer length of stay at the migration destination was associated with STIs among the Nepalese and Chinese migrants [20], [22] and older age was shown to be associated with STIs and HIV [22], [25]. Some of the variations between studies will be due to differences in study design, sampling, laboratory tests and how migrants are defined.

There are conflicting data for how either living with a spouse or length of stay at a migration destination are associated with higher risk of HIV and STI infection [5], [20], [22], [26], [27]. Other studies have assumed that men who migrated to a destination recently will have higher risk behaviour than men who migrated prior to this [14], [28], [29]. However we found that living with a wife or living in Surat for at least 10 years was associated with having HIV-1, although the numbers were small. Living in Surat for at least 10 years was also significantly associated with having an STI among our participants. In contrast, living in Surat for less than two years was not associated with either HIV-1 or STI infection. This suggests that migrant men who have resided in Surat for a longer duration may have more access to sex partners than men who have recently migrated there. We postulate that this may be due good employment prospects and income in Surat so that there is spare money available to spend on sexual partners. HIV/STI risks and behaviours may differ between migrants who have employment and migrants who do not.

Although the participants’ HIV prevalence was similar to ANC attendees in the 2005 sentinel surveillance (1.3%), a proxy group for Surat’s general population [9], this is three times greater than the 2006 national Indian general population HIV prevalence estimate (0.36%) [30]. The HIV prevalence among study participants is therefore likely to represent an increase in risk after migration since the majority (698) of the 845 study participants were from low HIV prevalence Indian states. The sentinel surveillance HIV prevalence in Surat has remained above 5% among STI clinic attendees and high risk groups and above 1% among ANC attendees since 2005 [31], [32], [33]. Commercial sex work is likely to have played a significant role in this especially following the closure of the red light area (RLA) in 2003which greatly hampered the implementation of a HIV/STI prevention programme to the sex workers.

Mathematical modelling based on Indian data suggests that male migration itself can lead to a change in sexual networks in their place of origin [34]. In a South African cohort study HIV acquisition in female partners of migrant men in the place of origin was independent of the men’s HIV status [7]. Further prospective studies are required to clearly understand the association between migration and HIV in populations at risk [35].

A substantial proportion of STIs were likely recently acquired STIs (38.9%) among participants and the increased risk of HIV acquisition and transmission due to the presence of STIs is well established [36], [37]. Workplace based HIV/STI prevention interventions were stopped in Surat in 2008 and replaced with residential interventions. The impact of this change is unclear however our study results together with the sentinel surveillance data from Surat support recommendations to reinstate workplace based interventions.

Similar to other studies of male migration in Asia, we found a lower prevalence of HIV and STIs among male migrants in our study compared to studies from Africa. Our findings challenge the concept that migrants are always at higher risk of HIV infection compared to the general population in the area to which they migrate, though the percentage of possibly recently acquired STIs is relatively high, indicating the risk for future, continued disease spread. Even if migration causes an increase in HIV and STI risk for individual migrants, the prevalence of infections in the areas of previous residence, the patterns of mixing between migrants and others, and the proportion of the population that are migrants all influence whether migrants will have a higher prevalence of infection than the general population in the area to which they migrated. Our findings suggest the need for data specific to a setting which can be contextualised with other local information to guide HIV/STI interventions.
